# Resonant Mixing in Glass Bowl Microbioreactor Investigated by Microparticle Image Velocimetry

**DOI:** 10.3390/mi10050284

**Published:** 2019-04-27

**Authors:** Sven Meinen, Lasse Jannis Frey, Rainer Krull, Andreas Dietzel

**Affiliations:** 1 Institute of Microtechnology, Technische Universität Braunschweig, 38124 Braunschweig, Germany; s.meinen@tu-bs.de; 2 Center of Pharmaceutical Engineering (PVZ), Technische Universität Braunschweig, 38106 Braunschweig, Germany; l.frey@tu-braunschweig.de (L.J.F.); r.krull@tu-braunschweig.de (R.K.); 3 Institute of Biochemical Engineering, Technische Universität Braunschweig, 38106 Braunschweig, Germany

**Keywords:** microbioreactor, femtosecond laser structuring, photosensitive glass, microparticle image velocimetry, capillary waves

## Abstract

Microbioreactors are gaining increased interest in biopharmaceutical research. Due to their decreasing size, the parallelization of multiple reactors allows for simultaneous experiments. This enables the generation of high amounts of valuable data with minimal consumption of precious pharmaceutical substances. However, in bioreactors of all scales, fast mixing represents a crucial condition. Efficient transportation of nutrients to the cells ensures good growing conditions, homogeneous environmental conditions for all cultivated cells, and therefore reproducible and valid data. For these reasons, a new type of batch microbioreactor was developed in which any moving mixer component is rendered obsolete through the utilization of capillary surface waves for homogenization. The bioreactor was fabricated in photosensitive glass and its fluid volume of up to 8 µL was provided within a bowl-shaped volume. External mechanical actuators excited capillary surface waves and stereo microparticle image velocimetry (µPIV) was used to analyze resulting convection at different excitation conditions in varied reactor geometries. Typical vortex patterns were observed at certain resonance frequencies where best mixing conditions occurred. Based on the results, a simplified 1D model which predicts resonance frequencies was evaluated. Cultivation of Escherichia coli BL21 under various mixing conditions showed that mixing in resonance increased the biomass growth rate, led to high biomass concentrations, and provided favorable growth conditions. Since glass slides containing multiple bowl reactors can be excited as a whole, massive parallelization is foreseen.

## 1. Introduction

Formulations of active pharmaceutical ingredients (APIs) are often only available in low amounts during the development phase. Therefore, comprehensive testing of their effect on cells in established screening tools, such as commercially available well plates, is often impossible. With the aim of solving this issue, new methods for screening based on microbioreactors (MBRs) were developed in the past [1,2,3,4]. Besides low consumption of precious API formulations, MBRs combine the advantages of fast mass transfer and the feasibility of parallelization [5,6]. Further miniaturizing MBRs down to few µL volumes would potentiate the advantages and more valuable screening data would be achievable without pushing up the costs.

MBRs which are open from the top with fluid volumes in the higher µL regime (>> 10 µL) are mostly based on commercial well plates. Microtiter plates are conventionally mixed via orbital shaking, ensuring good homogenization, because inertial and gravitational (volume) forces are dominating the capillary forces at these scales. However, with decreasing the volumes of MBRs to <10 µL, the volume forces rapidly reduce. Therefore, MBRs with few µL volumes are typically realized as closed systems where mixing is achieved by diffusion [2,7] or active convection through rising gas bubbles [8,9,10]. Closed MBRs have to be connected via tubes and, for example, filled by syringe pumps and cannot be filled like well plates by using automatic dispensers. Because of the tremendous increase in tubing and feeding channels, it is challenging to massively parallelize these systems as it is required for fast and parallel screening of the biological efficiency. Open and highly parallelized MBR systems with volumes below 10 µL can be found in the literature only in the form of hanging droplet cultivations without active convection which are mainly used for cultivating spheroids and 3D cell cultivations [11,12,13]. However, with sedimentation and formation of temperature and mass gradients, cultivation of cells in suspensions without convection is restricted in hanging droplets.

Even though actively mixed open-top MBRs with volumes below 10 µL cannot be found in literature, a promising concept for mixing fluids in this regime is given by the excitation of capillary waves. This is due to the fact that capillary forces are beginning to dominate, if the dimension of the microfluidic system is below the capillary length of water $l_{C}=\sqrt{\gamma \cdot {\left(g\rho \right)}^{-1}}=2.71\;\mathrm{mm}$ (with the gravitation constant g, the surface tension of water $\gamma =0.072\;N\cdot m^{-1}$, and the density of water ρ). Some nonbiologic microfluidic systems are already making use of surface effects in order to enhance mixing. Shilton et al. used surface acoustic waves to mix an open-top well with a volume of 2.5 µL and achieved mixing times of 5 to 10 s depending on the power input [14,15]. In addition, the authors found a correlation between fluid velocity in azimuthal plane and mixing time. Electrowetting was used for mixing a droplet (V≈2 µL) covered with silicone oil by Mugele et al. [16] and Oh et al. [17]. In both works, a droplet was forced to vibrate by applying an alternating current to a pair of electrodes. This led to capillary waves on the interface between water and oil and induced stationary vortices inside the droplet. Flow velocities of around $0.3\;\mathrm{mm}\cdot s^{-1}$ and mixing times under 10 s were achieved. In these works, the lateral fluid velocity ${\left|u\right|}_{\mathrm{lat}}$ is explained as a result of a lateral motion as first described by Stokes [18] and known as Stokes drift, which is a function of the distance z to the fluid surface and the amplitude of the capillary wave $\xi_{\max}$:

(1) $${\left|u\right|}_{\mathrm{lat}}=4\pi \lambda^{-1}\;\xi_{\max}^{2}\cdot e^{-\frac{2\pi z}{\lambda }}$$

Capillary waves are also described by Noblin et al. [19] to explain resonance modes of a droplet (V=100 µL) vibrated by a loud speaker. In their work, a 1D approximation was made to determine resonance wavelengths $\lambda_{n}$ as:

(2) $$\frac{n\lambda_{n}}{2}=L_{p}$$

with n=1,2,3,…∞ representing the resonance modes and $L_{p}$ representing the length of the linear surface profile of the sessile droplet. Furthermore, the corresponding resonance frequencies $f_{n}$ were calculated, adapting an equation of Landau et al. [20] as:

(3) $$f_{n}=\sqrt{\left(\frac{\pi n^{3}\gamma }{4\rho L_{p}^{3}}+\frac{\pi ng}{L_{p}}\right)\cdot \tanh\left(\frac{\pi nh}{L_{p}}\right)}$$

with h being the depth of the liquid bath. Their experiments revealed resonance frequencies slightly lower than the calculated ones. A marginally more complex equation, additionally considering the pressure differences inside the droplet, was set up by Kim et al. [21] in their work on vibrating droplets. In their experiment, the droplet (V≈7 µL) had a radius $R_{d}\ll l_{C}$, so that gravity could be neglected. However, resonance frequencies determined experimentally showed 10% deviation from their analytical model predictions. In the same work, the mode patterns were visualized using microparticle image velocimetry (µPIV). The authors identified vortex patterns comparable to those excited by electrowetting.

Besides mixing, MBRs are facing the challenge of monitoring essential data during the growth of cells. Because of the small volume, it is inevitable to directly implement all sensors. Using microtechnology, electrochemical [22] or optical sensors [7] can be fully integrated. Most micromanufactured MBRs are based on polydimethylsiloxane (PDMS) and fabricated by soft lithography. This technology is well known and cheap regarding the necessary equipment and the materials [5], but PDMS tends to adsorb molecules, shows limited stability in systems operated under high pressures, and is hydrophobic [23]. Micromanufacturing in glass requires more elaborate techniques. A complex lithographic process for 3D flow-focusing devices has been recently developed [24]. Moreover, femtosecond laser ablation processes were investigated to provide through-glass via in microelectromechanical systems (MEMS) [25] and transparent Taylor flow devices [26]. Even though laser ablation of glass has been demonstrated to overcome typical drawbacks of soft lithography with PDMS, restrictions with respect to the achievable aspect ratio and the free shape of the structures remain. With Laser Direct Writing (LDW) using femtosecond laser pulses in photosensitive glass such as commercially available Foturan^®^ (Schott AG, Mainz, Germany), true 3D systems with well-defined walls and embedded channels can be manufactured [27,28,29].

Here, novel glass bowl microbioreactors (GB-MBRs) manufactured by LDW in wafers of Foturan^®^ (Schott AG, Mainz, Germany), holding fluid volumes of 8 µL, are presented and the effectiveness of capillary-wave-induced convection will be studied in detail by using µPIV.

## 2. Materials and Methods

### 2.1. GB-MBR Microfabrication

The GB-MBR was manufactured by femtosecond (fs) LDW in Foturan^®^ glass (Schott AG, Mainz, Germany), applying a process adapted from Cheng et al. [27]. A laser micromachining workstation (microSTRUCT C; 3D Micromac AG, Chemnitz Germany) was used which was equipped with a ytterbium-doped potassium–gadolinium–tungstate (Yb:KGW) laser source (PHAROS, Light Conversion, Vilnius, Lithuania), emitting at a fundamental wavelength of 1028 nm. For the Foturan^®^ exposure, the second harmonic wavelength at 514 nm was used. The beam was focused through a ×20 objective with numeric aperture NA=0.8 (Nikon, Tokyo, Japan) onto a 1.3 mm thick 4-inch Foturan^®^ wafer. The objective lens could be moved in *z*- and the substrate table in x- and y-directions to scan according to a predefined 3D pattern. Microfabrication was carried out at a pulse frequency of 100 kHz, pulse length of 212 fs, and a scanning speed of $200\;\mathrm{mm}\cdot s^{-1}$ at an average pulse energy of 12 µJ. After laser exposure, the glass samples experienced a heat treatment, following the procedure given by the substrate manufacturer. The temperature was elevated from room temperature to $300\;{}^{\circ }C$ at a rate of $3\;{}^{\circ }C\cdot \min^{-1}$ and then to 500 °C at a rate of $2\;{}^{\circ }C\cdot \min^{-1}$ and stayed at $500\;{}^{\circ }C$ for one hour. Then, temperature was raised at $1\;{}^{\circ }C\cdot \min^{-1}$ to $600\;{}^{\circ }C$ where it remained for another hour before heating was switched off and the samples cooled down to room temperature. During heat treatment, a brownish crystalline phase was formed at the exposed areas. In the following step, this phase was etched in a stirred bath of 10% hydrofluoric acid (HF, Honeywell Fluka^TM^, Fisher Scientific GmbH, Schwerte, Germany) for about 2 h until no more crystalline material was seen. To achieve highly transparent devices, a post-treatment of the structures as described by [28] was applied. The surfaces were cleaned in 10% ${\mathrm{HNO}}_{3}$ (Merck KGaA, Darmstadt, Germany) with ultrasonic support to remove residual silver grains, which deposited on the surface during the HF etching. A tempering at 600 °C for 2 h led to smoothening of the surfaces. 

**Figure 1:**
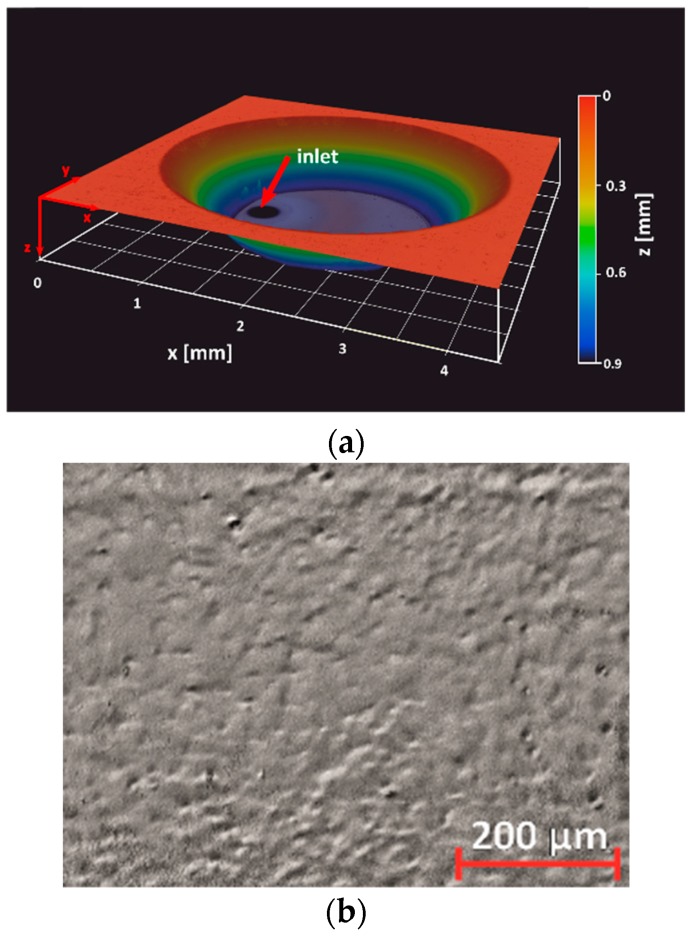
(**a**) 3D topography profile of a finished GB-MBR with microfluidic feed channel inlet (black circular area at the bottom) with entrance diameter of 350 µm, tapered to 150 µm (obtained with laser scanning microscopy). To facilitate imaging of tapered walls, the reactor was sputter-coated with a 25 nm thick layer of gold. (**b**) Laser scanning image of the GB-MBR’s bottom surface showing surface roughness ($r_{a}=0.16\;µm$).

**Figure 2:**
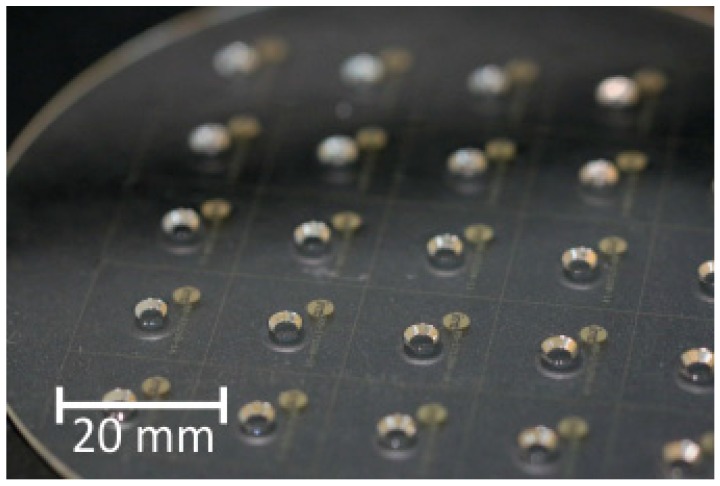
5×5  array of GB-MBRs manufactured on a 4 inch Foturan^®^ wafer.

To allow seeding bioprocesses with APIs, the GB-MBR was opened to the top. Further design requirements of the GB-MBR were high transparency, low surface roughness to ensure planktonic processing of cells, a volume of less than 10 µL, and a microfluidic channel, which shall in the final form of application be used to continuously feed the GB-MBR with water to compensate evaporation. All designs of the GB-MBR had a diameter at the top of 4 mm and a nominal depth of 1 mm but they differed in the steepness of the wall described by the wall slope S. Figure 1a shows a 3D topography profile of a single GB-MBR, whereas Figure 1b shows the surface of the bottom of a reactor where surface roughness $r_{a}$ was measured. An array of fabricated reactors on a 4-inch wafer can be seen in Figure 2.

The 3D profile (Figure 1a) obtained by laser scanning microscopy (VKX200, Keyence, Osaka, Japan) reveals a depth of 0.9 mm. The slight deviation of 0.1 mm from the nominal depth results from etching of the unexposed Foturan^®^ at the wafer surfaces. In addition, the measured wall slopes were slightly lower than defined by the design. Measured and nominal wall slopes of all designs are found in Table 1.

The deviation of nominal to measured S increases from 3.8% at $S=40{}^{\circ }$ to 6.8% at $\;S=60{}^{\circ }$. The difference can be explained by the thermal treatment. At higher temperature, the glass flows into the GB-MBR driven by gravitation. Moreover, steeper walls enhance this effect.

The contact angle of water $\theta_{C}$ on Foturan^®^ was measured optically with a drop shape analyzer (DSA 100E, A. Kruess Optronic GmbH, Hamburg, Germany). After laser treatment, annealing, HF etching, and ${\mathrm{HNO}}_{3}$ cleaning of glass surfaces, $\theta_{C}=29.4{}^{\circ }\pm 5{}^{\circ }$ was obtained. The observed high variance of the contact might be due to remaining surface roughness (Figure 1b), which was measured inside the GB-MBR as $r_{a}=0.16\;µm$ by laser scanning microscopy. This is far below the roughness of 1–3 µm given in the post-processing tolerances by the manufacturer [30]. 

### 2.2. Excitation of Resonant Mixing

**Figure 3:**
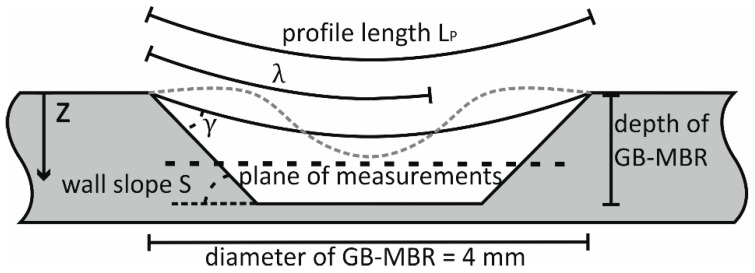
Schematic of GB-MBR cross-section. The diameter of the reactor is 4 mm, the nominal depth is 1 mm but was measured to be 0.9 mm only. Also shown are the contact angle γ, wall slope S, wavelength λ, profile length $L_{P}$, the plane where most PIV were done, and the direction of the *z*-axes.

For calculating resonances according to Equation (3) (neglecting gravity), the average depth of the fluid bath h and the profile length $L_{P}$ are needed. Both parameters are illustrated in Figure 3 and calculated considering the contact angle γ and the wall slope S. The model of Noblin et al. [19] assumes a fixed contact line between water and the substrate (or GB-MBR). Hence, for a noncompressible fluid, the mode n=1 is not possible, because it would imply a changing volume. Therefore, expected resonances would start with n=2.

For excitation, a voice coil actuator was used which was similar to an excitation setup described earlier [31]. The setup, however, was adapted to fit into the PIV measurement system. Instead of four, just one electrodynamic exciter (Ex 45 S, Visaton GmbH & Co KG, Haan, Germany) was used to periodically move the GB-MBR up and down (in z-direction). The exciter received the sinusoidal and amplified signal from a frequency generator (Agilent 33220A, Agilent Technologies, Santa Clara, USA; LF amplifier: Conrad Electronic SE, Hirschau, Germany). The resulting equation of motion of the GB-MBR and its first derivation can be described as:

(4) $$z\left(t\right)=z_{\max}\times \sin\left(2\pi f\times t\right)$$

(5) $$v\left(t\right)=\frac{dz\left(t\right)}{dt}=2\pi f\times \cos\left(2\pi f\times t\right)$$

**Figure 4:**
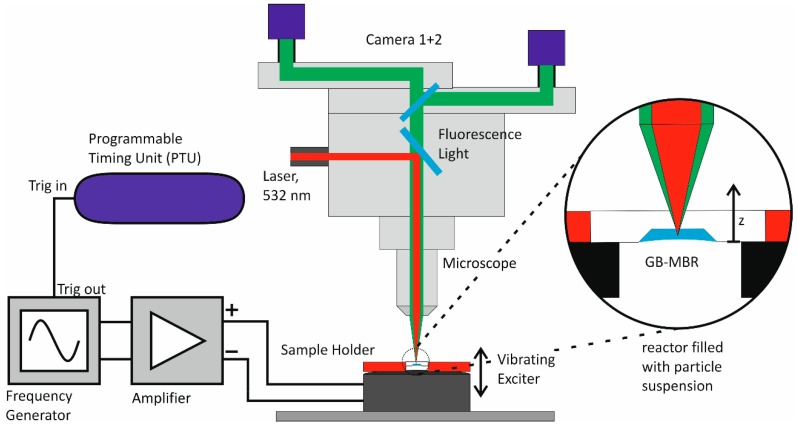
The stereoscopic µPIV setup with exciter and GB-MBR.

The maximum amplitude $z_{\max}$ depends on the settings of the frequency generator f and signal intensity $V_{pp}$. $z_{\max}$ was measured for the selected values ($f,\;V_{pp})$ by taking images of fluorescence particles placed on the moving GB-MBR only at times $\left(2/3+2N\right)\cdot \pi f^{-1}$ (with N being an arbitrary integer number) to measure at maximum deflection. The z-axis of the below-explained microscopic system (Figure 4) was adjusted until sharp particle images were seen. The result was a matrix filled with values $z_{\max}(f,V_{pp}$). For the rows (fixed f values), it was found that a linear fit gave good extrapolations for unmeasured values, and the columns (fixed $V_{pp}$.values) could be fitted with an exponential curve. These results are needed for the control of the experiments, ensuring that µPIV is always done at a defined z-position in the moving coordinate system of the GB-MBR (Figure 3).

To compare µPIV at different frequencies, they have to be executed at the same averaged volumetric power input $P_{\mathrm{mix}}$, which is given as:

(6) $$P_{\mathrm{mix}}=\frac{f}{V}\int_{0}^{2\pi }dW\left(t\right)dt=\frac{f}{V}\int_{0}^{2\pi }\frac{1}{2}m\cdot \frac{d{\left(v\left(t\right)\right)}^{2}}{dt}dt=\;4\rho \pi^{2}f^{3}z_{\max}^{2}$$

with W(t) representing the kinetic energy of the fluid volume, ρ the density, and V the volume of the fluid. The voice coil exciter can deliver harmonic response only in a limited regime. Therefore, at f=250 Hz, not more than $P_{\mathrm{mix}}\approx 300\;W\cdot m^{-3}$ is attainable, but at f=66 Hz, up to $P_{\mathrm{mix}}\approx 1500\;W\cdot m^{-3}$ can be reached. 

Assuming that observed capillary waves are forced vibrations of a harmonic oscillator, the location of the fluid elements ξ(t) and their velocity in z-direction w(t) are described by:

(7) $$\xi \left(t\right)=\xi_{\max}\cdot \sin\left(2\pi ft-\Delta \varphi \right)$$

(8) $$w\left(t\right)=\frac{d\xi \left(t\right)}{dt}=2\pi f\cdot \xi_{\max}\cdot \cos\left(2\pi ft-\Delta \varphi \right)$$

with $\xi_{\max}$ representing the wave height of the capillary wave. In steady state and resonance, the phase shift Δφ=π/2 occurs between the oscillation of the waves ξ(t) and the exciting sinusoidal oscillation z(t) (Equation (4)). Therefore, w(t) and z(t) are in-phase

(9) $$w\left(t\right)=2\pi f\frac{\xi_{\max}}{z_{\max}}z\left(t\right)$$

It follows that µPIV fields captured at $z_{\max}$ will reveal $w_{\max}$ and $\xi_{\max}$ is given as:

(10) $$\xi_{\max}=\frac{w_{\max}}{2\pi f}$$

Furthermore, for a harmonic oscillation in resonance, the relation between $\xi_{\max}$ and $z_{\max}$ is given with the damping of the waves b by [32]:

(11) $$\xi_{\max}=\frac{2\pi z_{\max}}{b}$$

The Stokes drift causes the lateral movement inside the GB-MBR (Equation (1)) and is proportional to $\xi_{\max}^{2}$ (Equation (10)). $\xi_{\max}$ is directly proportional to $z_{\max}$ (Equation (11)). Together with Equation (6) it follows:

(12) $${\left|u\right|}_{\mathrm{lat}}\sim \xi_{\max}^{2}\sim z_{\max}^{2}\sim P_{\mathrm{mix}}$$

The latter shows that increasing the volumetric power input proportionally increases the lateral movement and therefore the mixing inside the GB-MBR.

### 2.3. Particle Image Velocimetry

For image recording, a commercial µPIV system (stereo-Micro-PIV FlowMasterSystem, LaVision GmbH, Göttingen, Germany) was used. The exciter was placed underneath the microscope objective (PlanApo S, FWD 30 mm, Carl Zeiss AG, Oberkochen, Germany) as sketched in Figure 4. The GB-MBR was flipped for not disturbing the microscopic imaging by the oscillating water/air interface. Fluorescence seeding particles (Fluoro-Max^TM^, Thermo Fisher Scientific Inc., Waltham, USA) were used for obtaining flow velocity distributions. For fluorescence excitation, a double-pulse laser (Litron Bernoulli; Litron Ltd., Rugby, UK) with a wavelength of 532 nm was used, which was focused through the observation objective with a depth of focus DOF=170 µm. A filter ensured that no laser light was transferred to the cameras (Imager sCMOS, LaVision GmbH, Göttingen, Germany). With two optical paths and two independent cameras, stereo information was obtained.

The frequency generator triggers the cameras and the laser pulses. Images are always taken at the highest *z*-axis displacement of the GB-MBR. This leads to a frequency-dependent time gap dt between the images, varying from $dt_{\mathrm{start}}=1/f_{\mathrm{start}}=20\;\mathrm{ms}$ to $dt_{\mathrm{end}}=1/f_{\mathrm{end}}=4\;\mathrm{ms}$. It turned out that this range was acceptable for vector field calculations. A script (Visual Studio, Microsoft, Redmond, USA) was written to automatically take 20 double-frame images for each camera at increments of 2 Hz after waiting for 2 s to stabilize the flow. During the experiment, the microscope (SteREO Discovery.V20; Carl Zeiss AG, Oberkochen, Germany) was moved automatically in z-direction to match the focus to the changing highest z-displacement of the GB-MBR. Images were taken with focused plane at z≈0.6 mm (see Figure 3 and Figure 4). Measuring at lower z-positions leads to blurred images due to the particle suspension in the optical path. Vector calculations at these positions are possible, but not as reliable. Due to short duration of measurement, evaporation of liquid could be neglected. This experiment was performed for all different GB-MBR designs, taking a total of 4600 double-frame images with each camera for the calculation of 460 stereo vector fields. To investigate 3D-flow fields, images were also captured at different z-positons. Furthermore, taking images symmetrically before and after $z_{\max}$ with a small dt=100 µs eliminated the movement of the exciter but revealed $w_{\max}$ of the fluid elements at exemplary points.

Image processing and vector field calculations were performed using the software DaVis (LaVision GmbH, Göttingen, Germany). Before starting measurements, calibration of the system was done by taking images of a circle pattern with both cameras at two different focus positions of the microscope. An algorithm then calculated dewarped images for both cameras with the result that the coordinates from both cameras indicated the same positions. In detail, the calibration method is described by Calluaud and David [33]. All images were corrected using the shift and vibration correction offered by the software. This was necessary because the mechanical setup itself had specific resonances where vibrations with different frequencies emerged, leading to minor unintended movements of the reactor. Vector calculations were done by using sum of correlation and stereo cross correlation methods as proposed by Meinhart et al. [34] and implemented in DaVis. At first, a correlation map was calculated for each double-frame image. A correlation map showed for each interrogation window the shift in x and y, where first and second windows correlated best. Next, for the group of 20 double-frame images, the sum of all correlation maps was computed and then transformed into 2D vector fields (2D2C) by using the peak of highest correlation in each interrogation window. A vector field with three components in 2D (2D3C) was formed by comparing the results obtained from both cameras. 

### 2.4. Laser-Induced Fluorescence (LIF)

Using the same setup as described for µPIV, LIF images were taken in order to evaluate the mixing efficiency. The laser excites the fluorescent dye (Rhodamine B, Exciton, Lockbourne, USA) during a short pulse at high intensity. The following distribution of the dye is observed. Each experiment was prepared by filling the GB-MBR with 5 µL of water and carefully adding 1 µL of the dye with a pipette, trying not to mix the liquids in advance. Then, mixing and simultaneous image recording were started. Because images show the concentration of the dye, the uniformity is a measure for the degree of mixing.

### 2.5. Cultivation

To prove the applicability of the GB-MBR, cultivations with Escherichia coli BL 21 (DE3) were performed, because it is known that it is a fast-growing organism [35]. Accordingly, to prevent nutrient or oxygen gradients, a well-homogenized reaction volume is strongly required. Hence, fast growth of E. coli up to high cell densities is a good indicator for a well-mixed GB-MBR. Cultivation was performed using a lysogeny broth (LB) medium containing $10\;g\cdot L^{-1}$ tryptone, $5\;g\cdot L^{-1}$ yeast extract, and $5\;g\cdot L^{-1}$ sodium chloride (Carl Roth GmbH & Co. KG, Karlsruhe, Germany) [36]. The strain was transformed with a plasmid containing a Carbencillin resistance and the medium was supplemented with $100\;\mathrm{ng}\cdot L^{-1}$ Carbencillin. Inocula were precultured overnight in 100 mL shaken flasks (Schott AG, Mainz, Germany) filled with a volume fraction of 10% at $37\;{}^{\circ }C$, shaking speed of $120\;\min^{-1}$, and 50 mm shaking diameter (CERTOMAT IS, Sartorius Stedim Biotech GmbH, Göttingen, Germany). For an additional cultivation, 10 mL LB medium were inoculated with an ${\mathrm{OD}}_{600}$ of 0.1, using the overnight culture and applying similar cultivation conditions to increase the reproducibility of the cultivations. The second preculture was used subsequently to inoculate fresh medium in order to start the GB-MBR with an initial ${\mathrm{OD}}_{600}$ of 0.1.

Cultivations in the GB-MBR were performed in a cultivation chamber to adjust ambient humidity to 93% and temperature to $37\;{}^{\circ }C$ as previously described by Frey et al. [31]. Microbial growth was monitored by scattered light (SL) measurements, as frequently performed in literature [31,37,38,39]. Excitation was performed using a laser LED (AZ-Delivery Vertriebs GmbH, Deggendorf, Germany) with a maximum emission intensity at 656 nm connected to an optical fiber (FT400UMT, Thorlabs Inc., Newton, USA). Readout of SL was done at $90{}^{\circ }$ angle to excitation, using identical optical fibers connected to a spectrometer (USB2000+XR, Ocean Optics Inc., Largo, USA). Signals were processed and analyzed using the software Ocean View 1.6.7 (Ocean Optics, Ostfildern, Germany). Here, signals were integrated for 100 ms and 60 measuring points were averaged, resulting in a measuring frequency of $10{\;\min}^{-1}\;$.

## 3. Results and Discussion

### 3.1. Observation of Mixing by Laser-Induced Fluorescence (LIF)

**Figure 5:**
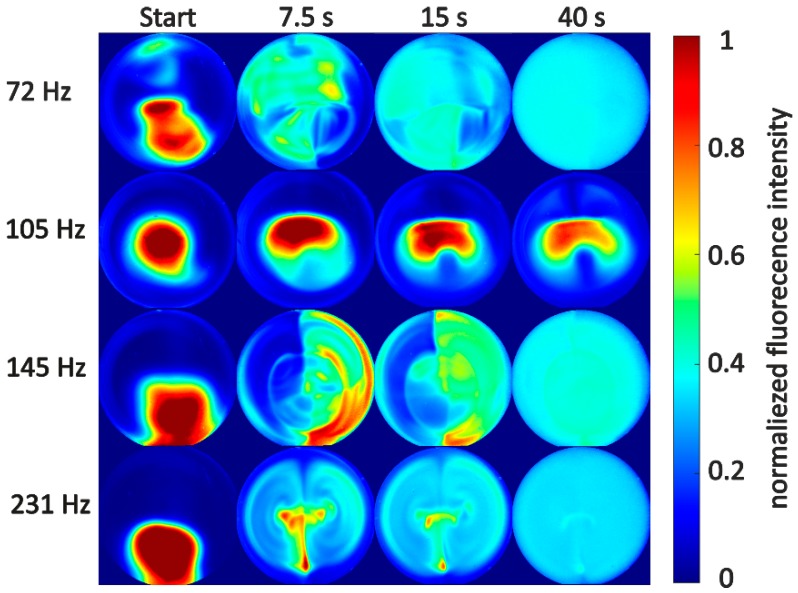
Exemplary LIF image series showing the normalized fluorescence intensity, indicating the qualitative concentration of 1 µL Rhodamine B during mixing in water within a GB-MBR with *S* = 50°. Shown image series correlates with frequencies calculated for the second, third, and fourth capillary wave resonances according to Equation (3). For comparison purposes, an image series for a frequency in between $f_{2}$ and $f_{3}$ is also shown. Volumetric power input $P_{\mathrm{mix}}$ was set to $300\;W\cdot m^{-3}$. From left to right, images are taken at 0, 7.5, 15, and 40 s after start of excitation.

In Figure 5, laser induced fluorescence images are shown at 0, 7.5, 15, and 40 s after the start of mechanical excitation. It only shows examples of the analysis and compares mixing behavior at frequencies for which a resonant response was expected with mixing behavior under nonresonant excitation. It is easily seen that faster mixing is achievable at resonance modes calculated according to Equation (3). At $f_{2}=72\;\mathrm{Hz}$, $f_{3}=145\;\mathrm{Hz}$, and $f_{4}=231\;\mathrm{Hz}$, complete mixing is achieved after 40 s, whereas at frequencies in between resonance modes (shown is an image series for an excitation frequency of 105 Hz), nearly no mixing is seen. 

The mixing at $f_{3}$ and $f_{4}$ seems to be driven by similar vorticity inside the GB-MBR: two vortices are formed, leading to mixing inside the GB-MBR. Similar vortices are described in other works investigating internal flow patterns induced by surface waves on droplets [14,15,16,17,40,41]. Looking at $f_{2}$, no clear vortex pattern can be identified but due to similar mixing times, it is assumed that also vortices drive the mixing. However, this analysis cannot prove whether mixing was investigated exactly at the real resonance frequencies, because calculations are only based on a simplified model. Analyzing mixing with LIF is associated with limitations: for each new set of parameters (changing frequency), the experiment has to be repeated with not easily reproducible initial fluorescent dye injection. Therefore, µPIV is used in the following to clarify the convection patterns inside the GB-MBR and to experimentally find exact resonance frequencies.

### 3.2. Convection Obtained by µPIV

**Figure 6:**
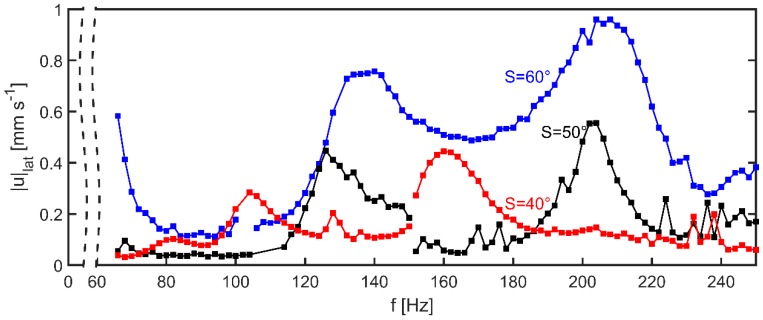
${\left|u\right|}_{\mathrm{lat}}$ measured inside reactors with different wall slope S in dependence of excitation. Because lower resonance modes are less intense, experiments at lower frequencies were performed with double volumetric power input of $600\;W\cdot m^{-3}$. The change between high and low input power is indicated as interruption of the line connecting data points (at 150 Hz for $S=40{}^{\circ }$ and $S=50{}^{\circ }$ and at 100 Hz for $S=60{}^{\circ }$). Experiments which are not being shown here are those with $S=55{}^{\circ }$ where similar results to $S=50{}^{\circ }$ were obtained as well as experiments with S=45 , leading to similar results as $S=40\;{}^{\circ }.$

Using MATLAB (MathWorks, Natick, USA, version R2017b), the amounts of local lateral velocity vectors $|u{|}_{j,i}$ were calculated for each vector in the vector field with the size $\left(i_{\max}\cdot j_{\max}\right)$ and the average ${\left|u\right|}_{\mathrm{lat}}$ over all vectors $|u{|}_{j,i}$ was calculated as:

(13) $${\left|u\right|}_{\mathrm{lat}}=\frac{1}{i_{\max}\;\cdot j_{\max}}\underset{i,j}{\sum }\sqrt{u_{x}{}_{i,j}^{2}+u_{y}{}_{i,j}^{2}}$$

to provide a measure for the convection intensity under different conditions (wall slope S of the GB-MBR, frequency f, volumetric power input $P_{\mathrm{mix}}$) at a certain z≈0.6 mm. Figure 6 displays the obtained values for ${\left|u\right|}_{\mathrm{lat}}$ as a function of excitation frequency between $f_{\min}=66\;\mathrm{Hz}$ and $f_{\max}=250\;\mathrm{Hz}.$ Differences between GB-MBRs with wall slopes between $S=40{}^{\circ }$ and $S=60{}^{\circ }$ are apparent. 

**Figure 7:**
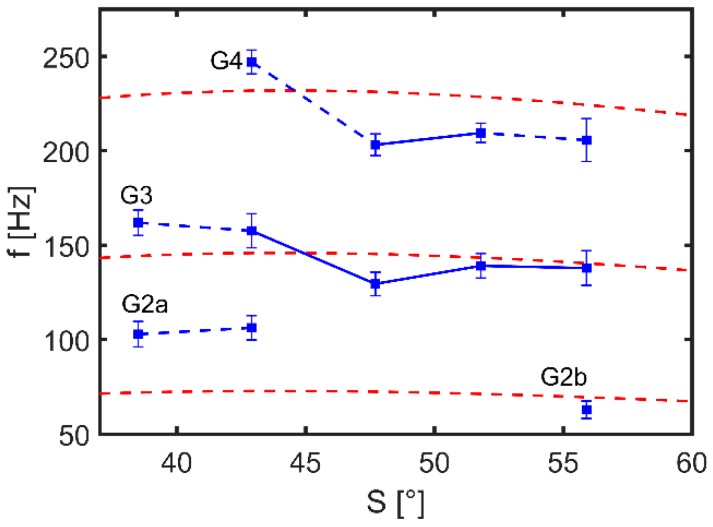
Experimentally determined resonance frequencies for GB-MBRs for different wall slopes S (blue) in comparison to resonance frequencies calculated with Equation 3 (red dashed lines). The resonance frequencies were determined as the positions of local maxima of ${\left|u\right|}_{\mathrm{lat}}$ determined by fitting a Gaussian normal distribution. The obtained standard deviations are given as error bars. Data points were grouped and named (G2 to G4) based on the associated resonance mode. Data points connected only with dashed lines indicate same resonance modes but different flow patterns.

At local maxima, velocities are in a range from 0.3 to $1\;\mathrm{mm}\cdot s^{-1}$, which is a comparable range to similar experiments with capillary waves actuated by electrowetting [16] and to numerical simulations of a droplet with a sinusoidal variation of the contact angle [17]. At lower values of f at local maxima, values of ${\left|u\right|}_{\mathrm{lat}}$ are lower but maxima can clearly be identified for all experiments. At lower wall slope S, the lateral flow seems to be hindered perhaps because of lower average depth of the bath. Because the upper diameter of all GB-MBRs is fixed to 4 mm, at flatter walls the average depth of the GB-MBRs is slightly lower. Moreover, this effect could be explained by the stronger curvature of the water meniscus in the GB-MBRs with higher S. Hence, the capillary waves have a larger lateral component in the amplitude, which could lead to a higher velocity. Data was further investigated by fitting Gaussian distributions to the peaks after background subtraction. For comparison, the resonance frequencies in dependence of *S* according to Equation (3) are indicated as dashed lines (Figure 7).

For all measurements, peaks close to the calculated frequency of the third resonance mode (G3) where found. Nevertheless, for lower wall slopes, a clear assignment to n=3 was not possible, because for $S=35{}^{\circ }$ and $S=40{}^{\circ }$, two maxima were recognized just above (G3) and below (G2a) the calculated values. The latter could also be associated with n=2. In all other GB-MBRs, no resonance at n=2 was found, probably because measurements were done only down to f=66 Hz which is close to the calculated resonance frequency $f_{2}$. For the three GB-MBRs with steeper walls, all other resonances were at frequencies somewhat lower than those given by the 1D model. 

Assuming that all peaks of G2a and G2b are associated with n=2, G3 with n=3, and G4 with n=4, the differences between model and experimental values are following the same trend: For $S=35{}^{\circ }$ and $S=40{}^{\circ }$, experimentally obtained $f_{n}$ was higher; for the others, $f_{n}$ was lower than the model. 

**Figure 8:**
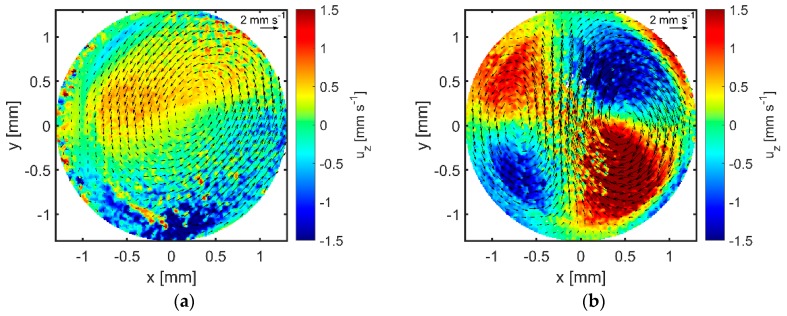
Typical single-vortex flow pattern obtained by µPIV in a GB-MBR with $S=60{}^{\circ }$ at $P_{\mathrm{mix}}=600\;W\cdot m^{-3}$ and (**a**) f=66 Hz and (**b**) f=206 Hz. The abscise and the ordinate indicate lateral coordinates *x* and *y* within the GB-MBR. The origin (0,0) is the center of the GB-MBR. The arrows indicate magnitude and direction of the lateral flow vector $u_{x},\;u_{y}$. The background color indicates magnitude and direction of $u_{z}$.

Only two distinct kinds of flow patterns were found. The first consisted of a single lateral vortex, the second of two vortices with inverse directions of rotation combining to a strong stream in the center of the GB-MBR. Exemplary patterns for both cases can be found in Figure 8.

Flow patterns with four symmetric vortices (not shown) occasionally appeared in the experiments. Mostly, this was the case at higher frequencies (f>200 Hz). At lower frequencies (f<100 Hz), not more than one vortex was formed. Therefore, a weak correlation between the maximum numbers of vortexes and f is recognizable. However, in the groups G3 and G4 shown in Figure 7, both, one or two vortexes can be found independent from parameters controlled by the setup. Mampallil et al. [42] also did not find any correlation between the resonance mode and the mode patterns in their electrowetting actuated droplet. However, they strongly manipulated the vortex pattern with the help of small pinning structures. Transferring their results to the GB-MBR, this could imply that even small obstacles, like particles or surface roughness, have a substantial influence on the flow pattern.

**Figure 9:**
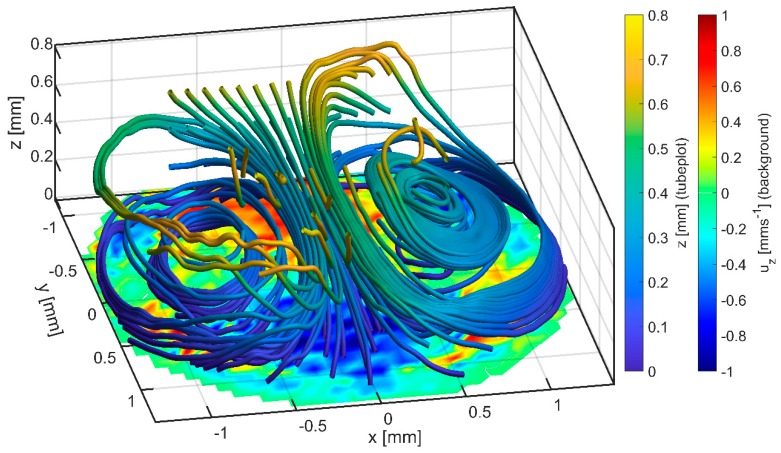
Tube plot showing the 3D velocity profile inside a GB-MBR. The color of the tubes indicates their location in z. At the bottom, $u_{z}$ is color-coded at the lowest measurable plane inside the reactor, which is close to the fluid surface. The data are generated by µPIV at different z-positions and are arranged into a 3D array containing velocity information at every point (x,y,z). The tube plot is generated using the function stream3() and tubeplot() from MATLAB: from user-defined starting points the tubes are following the 3D velocity field-like particles (of no mass). Hence, tubes have a start and an end, either given by the number of iterations or by the limits of the velocity field.

In addition to the flow patterns, magnitude and direction of the z-velocity $u_{z}$ are shown in Figure 8 by the color scheme. At the measured plane (z≈0.6 mm), the occurrence of a single vortex leads to a positive and negative $u_{z}$-region. For two vortices, two positive and two negative $u_{z}$-regions are observed. This indicates a warping of the circular movement in both cases. Further, to investigate this behavior, measurements at different z-heights were performed to capture most of the reactor volume in one 3D tube plot, which can be found in Figure 9. It is recognizable that the rotation axes of both vortices are slightly tilted towards the middle of the GB-MBR and the streamlines are bended, leading to mixing throughout the whole volume. Looking in positive *y*-direction onto the pattern, a vertical vortex would be recognizable too, although the lateral movement dominates.

To compare with the model of the Stokes drift, the values of ${\left|u\right|}_{\mathrm{lat}}$ measured at resonances in an GB-MBR with $S=60{}^{\circ }$ are given in Table 2 in comparison to values calculated by Equation (11) inserted in Equation (1) as:

(14) $${\left|u\right|}_{\mathrm{lat}}=\frac{n}{\pi f_{n}L_{p}}\;w_{\max}^{2}e^{-\frac{n\pi z}{L_{p}}};\;z=0.6\;\mathrm{mm}$$

For $f_{2}$ (66 Hz) and $f_{3}$ (138 Hz), the calculated values are of similar magnitude as the measured values. The strong difference at $f_{3}\;$(206 Hz) is probably because $w_{\max}$ was obtained from a measurement out of resonance. Additionally, the Stokes drift model suggests that ${\left|u\right|}_{\mathrm{lat}}\;\sim \;P_{\mathrm{mix}}$, which was validated by measuring lateral flows at different $P_{\mathrm{mix}}$. 

**Figure 10:**
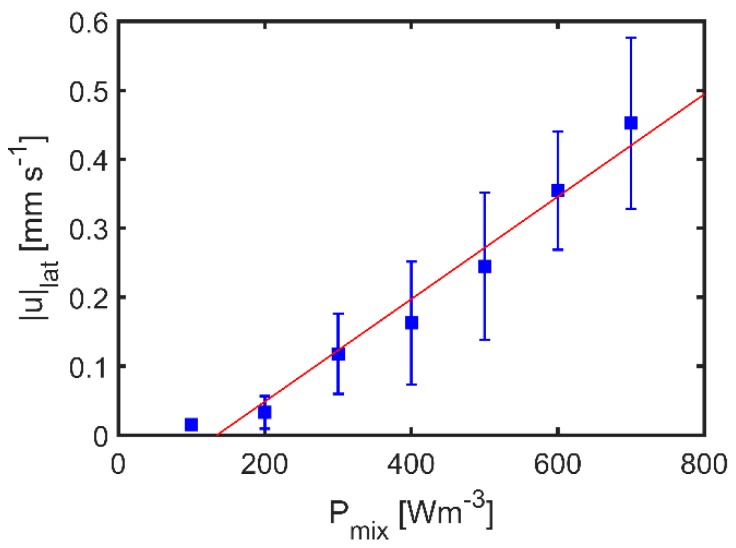
Lateral fluid velocity ${\left|u\right|}_{\mathrm{lat}}$ as a function of volumetric power input $P_{\mathrm{mix}}$ with error bars showing the standard deviation ±σ in a triplicate. Mixing was done in GB-MBR with $S=60{}^{\circ }$ at a frequency of f=148 Hz. The data were fitted by a linear function ${\left|u\right|}_{\mathrm{lat}}=m\cdot P_{\mathrm{mix}}-{\left|u\right|}_{0}$, with m being the proportionally constant and ${\left|u\right|}_{0}$ being the y-shift.

In the considered range, ${\left|u\right|}_{\mathrm{lat}}$ linearly depends on $P_{\mathrm{mix}}$ as shown in Figure 10. However, ${\left|u\right|}_{\mathrm{lat}}$ reaches zero already at $P_{\mathrm{mix}}\leq 170\;W\cdot m^{-3}$. At these low values of $P_{\mathrm{mix}}$, the damping of the system might suppress resonance and occurrence of the Stokes drift. The experiments show that a simple 1D model can predict resonance frequencies in the GB-MBR with about 10% accuracy. Further, the measured lateral flow velocities are in good accordance with the Stokes drift model. At higher order resonances, lateral flows intensify and therefore better mixing is expected.

### 3.3. Cultivations of Escherichia coli as Proof of Concept

**Figure 11:**
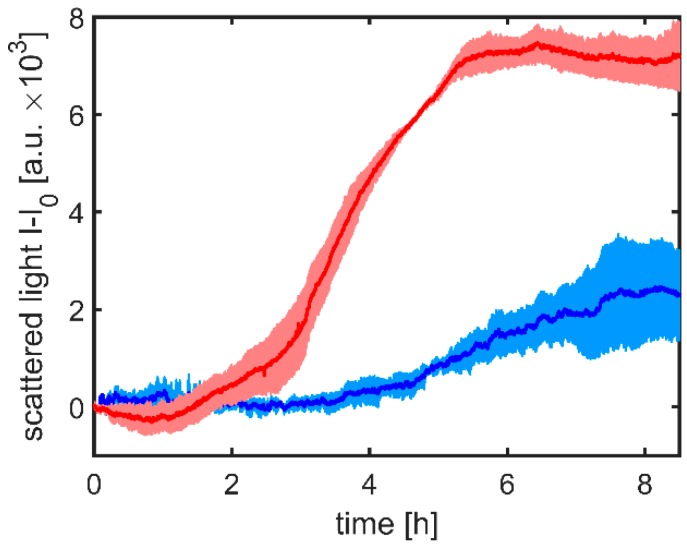
Cultivation of E. coli BL 21 in the GB-MBR mixed out of resonance (210 Hz, blue line) in comparison with resonant conditions (170 Hz, red line). As shown by SL, cells do grow marginally out of resonance. However, by adjusting to the resonance frequency, cell growth is substantially increased. The depicted curves are mean values of triplicates (n=3).

The cultivation of E. coli BL21 (DE3) was performed in the developed GB-MBR to prove its applicability as bioreactor. From µPIVs shown in Figure 6, highest lateral flows were identified at fourth resonance in the GB-MBR with $S=60{}^{\circ }.$ The change from pure water to LB medium has to be considered when predicting resonance frequencies. Rühs et al. [43] reported a surface tension $\gamma_{lb}\approx 0.045\;N\cdot m^{-}{}^{2}$ for the LB medium. Because it is an aqueous fluid, the changes in density are neglectable. Using Equation (3), a resonance of $f_{4}\approx 185\;\mathrm{Hz}$ was calculated. Because calculated resonances were always somewhat higher than the measured ones, the cultivation was homogenized with f=170 Hz. For comparison also out of resonance, measurements at f=210 Hz were performed at the same $P_{\mathrm{mix}}$ off $300\;W\cdot m^{-3}$. To monitor the course of microbial growth, SL measurements were monitored online and normalized on their initial value as previously described in literature [31,38]. Results are shown in Figure 11. The depicted curves are mean values of triplicates (n=3). Cultivations were started with an initial ${\mathrm{OD}}_{600}$ of 0.1. The cultivations out of resonance showed for the first 4 h almost constant SL intensities. After this rather long lag phase, cells started to grow for around 3.5 h to stop subsequently growing after 7.5 h of cultivation, reaching SL of around 3000 a.u. (arbitrary units). In contrast, SL of the cultivations mixed in resonance started to increase just after 1 h. Subsequently, the exponential growth was observed for about 4 h, which then rapidly transitioned to a stationary phase that lasted for the final 4 h of the cultivation. Here, SL values of more than 7000 a.u. could be measured.

For cultivation, parameters out of resonance cells do grow marginally, but by adjusting the resonance frequency, cell growth can be substantially accelerated and increased. Hence, oscillation in resonance shortens the lag phase distinctly, increases the biomass growth rate, and leads to considerably higher biomass concentrations. Consequently, mixing in resonance constitutes clearly more favorable growth conditions for the here applied bacteria. Even if mixing times found in LIF experiments could still be shortened with higher power input, the resonant excitation of capillary waves is thus a proven and suitable possibility to homogenize the GB-MBR.

## 4. Conclusions

In present investigations, a new type of MBR was successfully developed in the form of an open-top glass bowl named GB-MBR holding liquid volumes of below 10 µL. It was microfabricated in photosensitive Foturan^®^ glass by exposure with femtosecond laser beams, followed by a subsequent procedure with thermal treatment and KOH etching. As a result, transparent GB-MBRs with smooth hydrophilic walls ($r_{a}=0.16\;µm,\;\theta_{C}=29.4{}^{\circ }$) were obtained. The GB-MBR was homogenized using capillary waves excited by a voice coil actuator, showing a resonant behavior with strong internal convection at certain frequencies. By using stereoscopic µPIV, the induced convection was studied in depth; typical vortex patterns could be identified and clearly illustrated in a 3D tube plot. Lateral convection as obtained by µPIV was strongly dependent on the excitation frequency, with maxima at distinct resonance frequencies. Using a simplified 1D model for capillary waves, resonance frequencies could be approximated in a sufficiently precise manner based on the GB-MBR geometries. Experimentally observed lateral convection in dependence of the amplitude of capillary waves was in good agreement with predictions assuming Stokes drift. Different geometric variations were studied which led to variations in achievable maximum lateral velocities. A steeper wall slope was found to be advantageous regarding the strength of the internal convection.

For a first biological proof of concept, a batch cultivation of E. coli BL 21 was carried out in the GB-MBR. It could be observed that under resonant conditions, the cell proliferation was much faster and final biomass concentration was nearly four times as high as for nonresonant excitation. The results are encouraging for doing experiments with mammalian cells in future, which shall be used to test special formulations of APIs. In future research works, we will also investigate the integration of sensors in glass and the compensation of evaporation through small replenishment channels. The concept of laser fabrication on wafer level easily allows producing larger arrays of GB-MBRs for fast parallel screening. When combined with robotic pipetting, highly parallelized GB-MBRs are a very promising approach for fully automated screening of active pharmaceutical components in high-throughput manner.

## Figures and Tables

**Table 1:** Nominal wall slope S given by CAD in comparison to measured values.

$\mathrm{Nominal}\;S\;({}^{\circ })$	$\mathrm{Measured}\;S\;({}^{\circ })$	Deviation (%)
40	38.5	3.8
45	42.9	4.7
50	47.7	4.6
55	51.8	5.8
60	55.9	6.8

**Table 2:** Experimentally obtained ${\left|u\right|}_{\mathrm{lat}}$ compared to values calculated using the model of the Stokes drift. Measured values ${\left|u\right|}_{\mathrm{lat}}$ are the same as in Figure 6, $S=60{}^{\circ }$. $w_{\max}$ was determined by µPIV as described in the methods and taking a local average of z-velocities around the maximum z-velocity found in the vector field.

Mode n	$f_{n}$ (Hz)	$|u{|}_{\mathrm{lat}}\;$ $\mathrm{Measured}\;(\mathrm{mm}\cdot s^{-1})$	$|u{|}_{\mathrm{lat}}\;$ $\;\mathrm{Calculated}\;(\mathrm{mm}\cdot s^{-1})$
**2**	66	0.58	0.31
**3**	138	0.76	0.55
**4**	206	0.95	0.04
